# The quest for load‐independent left ventricular chamber properties: exploring the normalized pressure–volume loop

**DOI:** 10.14814/phy2.13160

**Published:** 2017-03-29

**Authors:** Keshav Kohli, Sándor J. Kovács

**Affiliations:** ^1^ Cardiovascular Biophysics LaboratoryCardiovascular Division Department of MedicineWashington University School of MedicineSt. LouisMissouri; ^2^ Department of Electrical EngineeringSchool of Engineering and Applied ScienceWashington University in St. LouisSt. LouisMissouri; ^3^ Department of Biomedical EngineeringSchool of Engineering and Applied ScienceWashington University in St. LouisSt. LouisMissouri

**Keywords:** Catheterization, hemodynamics, load‐independence, pressure–volume loops

## Abstract

Left ventricular (LV) pressure–volume (*P*–*V*) loop analysis is the gold standard for chamber function assessment. To advance beyond traditional *P–V* and pressure phase plane (d*P*/dt‐P) analysis in the quest for novel load‐independent chamber properties, we introduce the normalized *P*–*V* loop. High‐fidelity LV pressure and volume data (161 P‐V loops) from 13 normal control subjects were analyzed. Normalized LV pressure (*P*_N_) was defined by 0 ≤ *P*(t) ≤ 1. Normalized LV volume (*V*_N_) was defined as *V*_N_=*V*(*t*)/*V*_diastasis_, since the LV volume at diastasis (*V*_diastasis_) is the in‐vivo equilibrium volume relative to which the LV volume oscillates. Plotting *P*_N_ versus *V*_N_ for each cardiac cycle generates normalized P‐V loops. LV volume at the peak LV ejection rate and at the peak LV filling rate (peak −d*V*/dt and peak +d*V*/d*t*, respectively) were determined for conventional and normalized loops. *V*_N_ at peak +d*V*/dt was inscribed at 64 ± 5% of normalized equilibrium (diastatic) volume with an inter‐subject variation of 8%, and had a reduced intra‐subject (beat‐to‐beat) variation compared to conventional P‐V loops (9% vs. 13%, respectively; *P* < 0.005), thereby demonstrating load‐independent attributes. In contrast, *V*_N_ at peak −d*V*/dt was inscribed at 81 ± 9% with an inter‐subject variation of 11%, and had no significant change in intra‐subject (beat‐to‐beat) variation compared to conventional P‐V loops (17% vs. 17%, respectively; *P* = 0.56), therefore failing to demonstrate load‐independent tendencies. Thus, the normalized P‐V loop advances the quest for load‐independent LV chamber properties. *V*_N_ at the peak LV filling rate (≈sarcomere length at the peak sarcomere lengthening rate) manifests load‐independent properties. This novel method may help to elucidate and quantify new attributes of cardiac and cellular function. It merits further application in additional human and animal physiologic and pathophysiologic datasets.

## Introduction

The pressure–volume loop (PVL) is widely used in cardiovascular physiology and cardiology for the assessment of cardiac chamber function. PVL analysis is the “gold standard” for left ventricular (LV) chamber function assessment, since it facilitates characterization of the load‐dependence versus load‐independence of various physiologic measures in humans and animals (Suga et al. 1973; Cingolani and Kass 2011). To generate a PVL for one (or multiple) heartbeat(s), high fidelity LV pressure (*P*) and volume (*V*) data are plotted against each other in the pressure–volume (*P–V*) plane, eliminating time as the explicit variable. The PVL characterizes cardiac mechanics during all four phases of the cardiac cycle: isovolumic relaxation, filling, isovolumic contraction, and ejection (Kass et al. 1986; Pacher et al. 2008).

Suga et al. (1973) pioneered the familiar conceptual framework for quantitative assessment of LV chamber properties by analyzing hemodynamics in the *P–V* plane. They observed that peak systolic elastance (*E*_max_), the (nearly constant) linear slope of the end‐systolic *P–V* relationship within a reasonable physiologic range, was indeed load‐independent at a fixed inotropic state (Suga et al. 1973; Kass et al. 1989). The meaning of load‐independence has been further characterized by the mathematical modeling of time‐varying elastance in kinematic terms (Oommen et al. 2003), leading to the first analytic proof that in‐vivo elastance could be a load‐independent index of contractility. Additional load‐independent LV chamber attributes have been characterized by Ghosh and Kovács (2013) by normalizing the pressure phase plane such that 0 ≤ *P*(*t*) ≤ 1 and −1 ≤ d*P*/dt ≤ 1. They observed that peak −d*P*/dt, corresponding to the peak rate of cross‐bridge dissociation during isovolumic relaxation, is inscribed very close to 61% of the peak pressure of the previous cardiac cycle. This was found to be *independent* of the peak pressure of the previous cycle (i.e., load‐independent).

To advance the methodologic quest for ‘new’ physiology, we present a *new method* of *P*–*V* data analysis for the determination of load‐independent LV chamber properties and propose an interpretation of its physiologic meaning. We introduce the dimensionless, normalized pressure–volume loop (nPVL). Pressure is normalized and becomes dimensionless by scaling the range of LV pressure oscillation to 0 ≤ *P*(*t*) ≤ 1. Volume is normalized and becomes dimensionless by scaling time‐varying volume relative to the equilibrium (diastatic) volume such that the normalized volume *V*_N _= 1 at diastasis. In this analysis, we focus on the value of normalized (dimensionless) volume during the peak rate of early diastolic filling (peak +d*V*/dt, corresponding to the Doppler E‐wave peak) and, by symmetry, during the peak rate of ejection (peak −d*V*/dt). We hypothesize that the nPVL method can provide insights into the load‐dependence versus load‐independence of LV chamber properties.

## Materials and Methods

### Subject population

Thirteen datasets were selected from our Cardiovascular Biophysics Laboratory database of simultaneous echocardiographic and high fidelity hemodynamic recordings (Lisauskas et al. 2001). Clinical characteristics are listed in Table 1. Each study subject provided signed, informed consent for participation in accordance with the Institutional Review Board (Human Research Protection Office) of Washington University School of Medicine. All subjects had elective cardiac catheterization at the request of a referring cardiologist to assess the presence or absence of potential coronary artery disease. Inclusion criteria for this study were the following: normal LV ejection fraction, normal sinus rhythm, normal valvular function, normal wall motion, and the presence of diastasis.

**Table 1:** Subject characteristics (*n* = 13)

Parameter	Value
Age, yrs	60 ± 11
Sex	
Male	62 (8/13)
Female	38 (5/13)
Height, cm	175 ± 10
Weight, lb	196 ± 37
Body mass index, kg/m^2^	29 ± 5
Heart rate, beats/min	68 ± 14
Hypertension	69 (9/13)
Ejection fraction, %	75 ± 6
No. of cardiac cycles	12 ± 3

Values are mean ± SD or % (*n*/*N*).

### Data acquisition

Our method for high fidelity, simultaneous LV pressure and volume recording has been described previously (Chung and Kovács 2008; Shmuylovich and Kovács 2008; Ghosh and Kovács 2012). In brief, simultaneous LV pressure and volume signals were acquired using a 6‐F triple transducer pigtail‐tipped P‐V conductance catheter (SSD‐1034; Millar Instruments, Houston, TX). Signals were calibrated using standard transducer control units (TC‐510; Millar Instruments). The aortic valve was crossed using fluoroscopy and the catheter was advanced toward the left ventricular apex, taking care to find a position that did not generate ectopic beats to assure stable hemodynamic recording. Pressure and volume signals were fed into clinical monitoring systems (Quinton Diagnostics, Bothell, WA or GE Healthcare, Milwaukee, WI) and a custom personal computer via a research interface (Sigma‐5DF; CD Leycom, Zoetermeer, The Netherlands) at a sampling rate of 250 Hz. LV ejection fraction was calculated from a calibrated ventriculogram (33 mL of contrast at 11 mL/sec through a 6‐F pigtail catheter (Cordis Corporation, NJ)) whose end‐systolic and end‐diastolic volumes were used to calibrate the volume channel.

### Derivation of normalized LV pressure (*P*_N_)

Our method for normalizing high fidelity LV pressure data has been previously described by Ghosh and Kovács (2013). We normalized LV pressure for each cardiac cycle according to:

$$P_{N}\left(t\right)=\frac{\left(P\left(t\right)-P_{\min}\right)}{\left(P_{\max}-P_{\min}\right)}$$

Such that *P*_min _= 0 and *P*_max _= 1 after normalization. As noted by Ghosh and Kovács (2013), this method of pressure normalization has revealed that *P*_N_ at the peak rate of cross‐bridge dissociation during LV isovolumic relaxation has load‐independent attributes.

### Derivation of normalized LV volume (*V*_N_)

The LV chamber is a mechanical suction pump with extracellular and intracellular elastic elements which, when modeled like springs, are loaded during systole and generate elastic recoil during diastole (Sonnenblick et al. 1986). In analogy to a spring, the oscillatory LV chamber also has a quantifiable equilibrium (kinematically static) state; however, since the LV is a three‐dimensional volume, its equilibrium state is also a volume. The LV volume at diastasis (*V*_diastasis_) was formalized by Shmuylovich et al. (1986) as the physiologic in‐vivo equilibrium volume. At physiologic equilibrium, the LV chamber is momentarily static and there is no flow. During this moment, the LV pressure and volume are constant and all forces on the chamber are balanced (they are not zero). Since the LV end‐systolic volume is always below the in‐vivo equilibrium (diastatic) volume, LV filling is always initiated by mechanical suction (d*P*/d*V* < 0). This suction is powered by stored elastic strain that generates motion as a result of cross‐bridge uncoupling (Shmuylovich et al. 2009). By appreciating that the LV is an oscillatory ‘volume pump’, it is self‐evident that the LV oscillates relative to *V*_diastasis_ with each cardiac cycle. Thus, to account for volume loading effects, we normalized LV volume with respect to the physiologic in‐vivo equilibrium volume (*V*_diastasis_) for each cardiac cycle according to:

$$V_{N}\left(t\right)=\frac{V(t)}{V_{\mathrm{diastasis}}}$$

**Figure 1:**
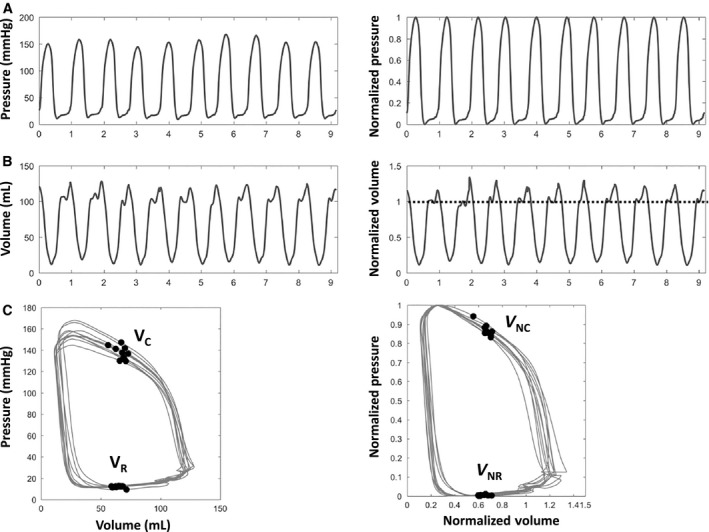
LV pressure–volume normalization method. Simultaneous high fidelity LV pressure and volume data is shown. (A) LV pressure (left panel), normalized as 0 ≤ *P*(t) ≤ 1 (right panel). (B) LV volume (left panel), normalized as *V*_N_(t) = *V*(t)/*V*_diastasis_ (right panel). (C) P‐V loop (left panel), normalized P‐V loop (right panel). Dotted line in B denotes the normalized equilibrium (diastatic) volume. *V*_C_ = volume at peak ejection rate; *V*_NC_ = normalized volume at peak ejection rate; *V*_R_ = volume at peak filling rate; *V*_NR_ = normalized volume at peak filling rate. See text for details.

Such that *V*_N_ = 1 at diastasis. Figure 1 summarizes the pressure–volume normalization method.

### Hemodynamic data analysis

**Figure 2:**
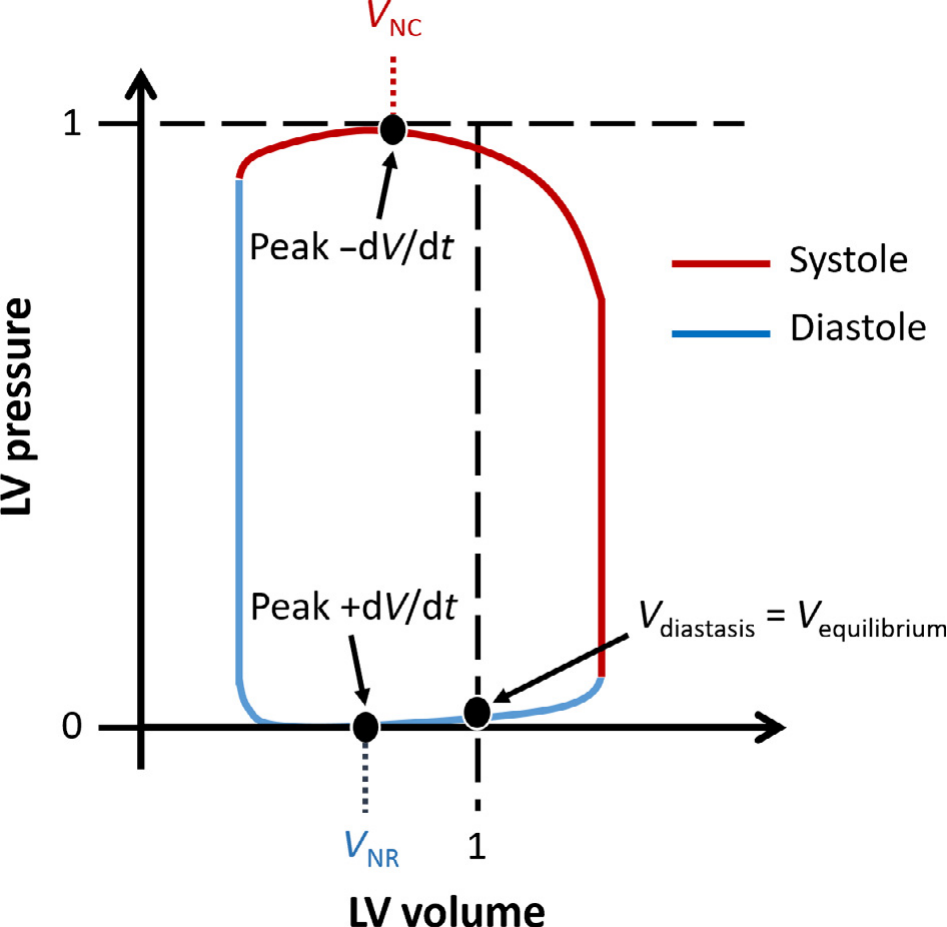
Conceptual framework for pressure–volume loop normalization. Volume is normalized relative to the equilibrium (diastatic) volume, such that *V*(t)/V_diastasis_  = 1 at diastasis. Pressure is normalized as 0 ≤ *P*(t) ≤ 1. Peak −d*V*/d*t* corresponds to peak aortic ejection (inscribed at V_NC_), and peak +dV/dt corresponds to the Doppler E‐wave peak (inscribed at V_NR_). See text for details.

**Figure 3:**
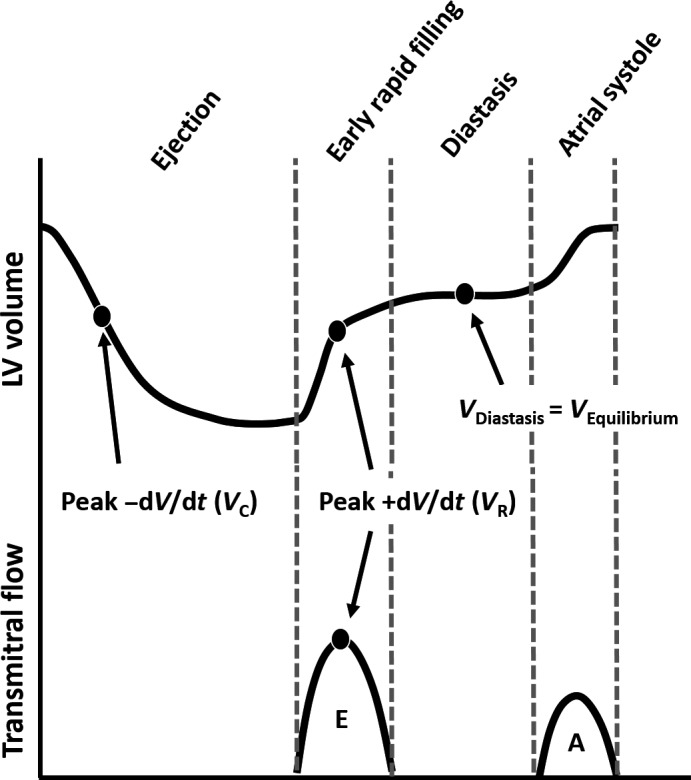
Correlation of LV volume parameters to transmitral Doppler flow velocities. Peak +dV/dt occurs at the same instant as the peak of the Doppler E‐wave. The LV volume at peak +d*V*/dt is denoted V_R_. Peak −dV/dt occurs during LV ejection. The LV volume at peak –d*V*/dt is denoted V_C_. See text for details.

High fidelity, simultaneously recorded LV pressure and volume signals were converted for quantitative analysis using a custom Matlab program (Matlab 6.0; MathWorks, Natick, MA). Plotting *P*_N_(*t*) versus *V*_N_(*t*) for each cardiac cycle generated nPVLs. Figure 2 outlines the key features of the nPVL. For all subjects, the following hemodynamic parameters were acquired and computed: *V*_diastasis_, volume at peak +dV/dt (*V*_R_), volume at peak −dV/dt (*V*_C_), normalized *V*_R_ (V_NR_), normalized *V*_C_ (V_NC_), *P*_max_, and *P*_min_. A custom Matlab program was used to calculate temporal derivatives for LV volume. Figure 3 illustrates the relationship between LV volume features and Doppler echocardiographic transmitral flow metrics.

### Statistical analysis

Statistical analysis utilized Matlab and Excel 2013 (Microsoft, Redmond, WA). The mean, standard deviation (SD), maximum and minimum values were calculated for all parameters of interest for both the PVL and nPVL (Tables 2, 3). Additionally, the coefficient of variation was calculated as the ratio of the standard deviation to the mean value, and is expressed as a percentage. The paired Student's *t*‐test was used to test for differences between intra‐subject (beat‐to‐beat) variation in parameters of interest in the PVL and nPVL.

**Table 2:** Group values (*n* = 13) of hemodynamic parameters

Parameter	Value
V_diastasis_, mL	97 ± 15 (76, 131)
Peak +dV/dt, mL/sec	2.6 ± 1.0 (0.97, 4.5)
Peak ‐dV/dt, mL/sec	−2.3 ± 1.1 (−4.7, −0.79)
Mean arterial pressure, mmHg	90 ± 13 (71, 112)
LV max. systolic pressure, mmHg	142 ± 26 (100, 175)
LV end‐diastolic pressure, mmHg	16 ± 5 (5, 24)
LV end‐systolic volume, mL	32 ± 8 (16, 45)
LV end‐diastolic volume, mL	127 ± 17 (104, 161)

Values are mean ± SD (min, max).

**Table 3:** Intra‐ and inter‐subject analysis of conventional and normalized *P–V* loop parameters

	Volume at peak +d*V*/dt (Doppler E‐wave peak)			Volume at peak −d*V*/dt		
	Conventional (*V*_R_, mL)	Normalized (V_NR_, dimensionless)	*P*‐value	Conventional (*V*_C_, mL)	Normalized (*V*_*NC*_, dimensionless)	*P*‐value
Value	63 ± 10 (44, 83)	0.64 ± 0.05 (0.57, 0.78)		78 ± 13 (55, 107)	0.81 ± 0.09 (0.65, 0.94)	
Intra‐subject (beat‐to‐beat) variation (%)	13 ± 6 (6, 27)	9 ± 5 (3, 21)	0.0024	17 ± 9 (6, 31)	17 ± 10 (4, 36)	0.56
Inter‐subject variation (%)	16	8		17	11	

Values are mean ± SD (min, max). Intra‐ and inter‐subject coefficients of variation are represented as %.

## Results

Because our intent was the assessment of a new method of PVL analysis, subjects were specifically selected to have normal LV function. Baseline demographic and clinical variables are listed in Table 1. All hemodynamic parameters of interest were calculated using both the conventional and normalized PVL methods and are summarized in Tables 2, 3. Normalization of the volume axis in the PVL reduced the variation in *V*_diastasis_ to 0 by definition. Additionally, normalization of the pressure axis reduced the variation in *P*_max_ and *P*_min_ to 0 by definition. Table 4 includes per‐subject parameters of heart rate, blood pressure, and number of cardiac cycles analyzed.

**Table 4:** Per‐subject hemodynamic values

Subject	No. of cardiac cycles	Heart rate, beats/min	Blood pressure, mmHg
1	9	55	166/64, 101
2	15	62	98/60, 72
3	8	58	150/88, 99
4	15	65	145/72, 91
5	15	66	125/60, 71
6	15	56	145/74, 88
7	15	66	122/77, 87
8	12	68	126/62, 74
9	6	89	161/90, 108
10	12	100	128/79, 93
11	15	50	142/66, 82
12	9	82	157/98, 112
13	15	70	160/82, 96

Per‐subject values are represented. Blood pressure is systolic/diastolic, mean.

The normalized LV volume at the peak rate of chamber filling (*V*_NR_) demonstrated load‐independent attributes, and was inscribed at 64 ± 5% relative to the normalized equilibrium (diastatic) volume for the thirteen subjects studied. The inter‐subject variation of *V*_R_ (16%) decreased to *V*_NR_ (8%) after normalization, demonstrating a reduction in inter‐subject variation with normalization. The normalized LV volume at the peak rate of ejection (*V*_NC_) was inscribed at 81 ± 9% relative to the normalized equilibrium (diastatic) volume. The inter‐subject variation of *V*_C_ (17%) decreased to *V*_NC_ (11%) after normalization. Among all studied parameters, *V*_NR_ had the lowest overall inter‐subject variation (Table 3). Normalization also decreased intra‐subject (beat‐to‐beat) variation of *V*_R_ (13 ± 6%) to *V*_NR_ (9 ± 5%) with statistical significance (*P* = 0.0024). However, the intra‐subject variation of *V*_C_ demonstrated no statistically significant change compared with *V*_NC_ (17 ± 9% vs. 17 ± 10%, respectively; *P* = 0.56).

**Figure 4:**
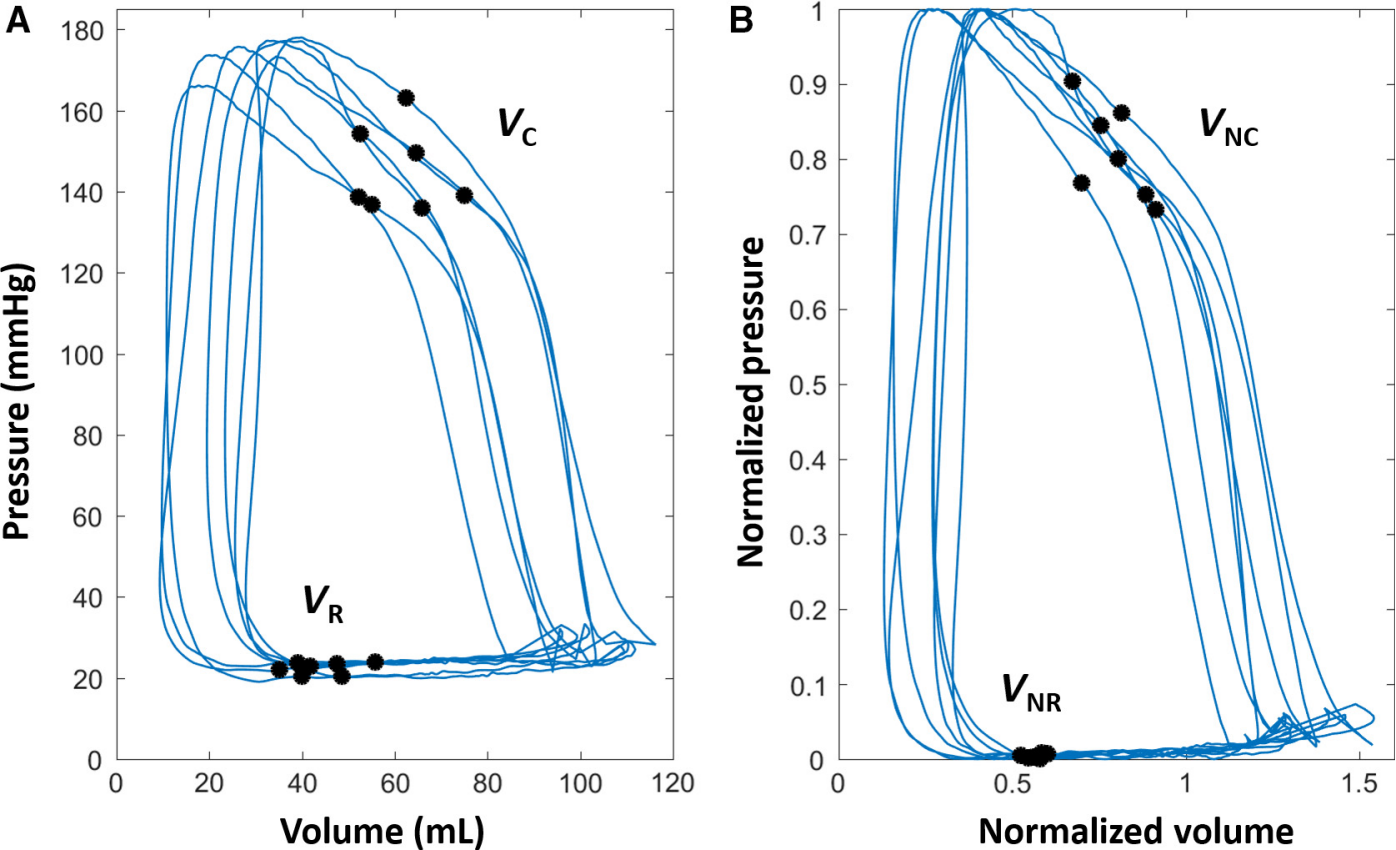
Convergence of intra‐subject (beat‐to‐beat) variation of V_R_ with *P–V* loop normalization. Conventional (A) and normalized (B) P‐V loops for a selected subject are shown. The small intra‐subject (beat‐to‐beat) variation in V_NR_ (i.e., normalized *V*_R_) indicates that the volume at the peak rate of chamber filling (i.e. peak +d*V*/dt = Doppler E‐wave peak) relative to the diastatic volume exhibits load‐independent tendencies. See text for details.

**Figure 5:**
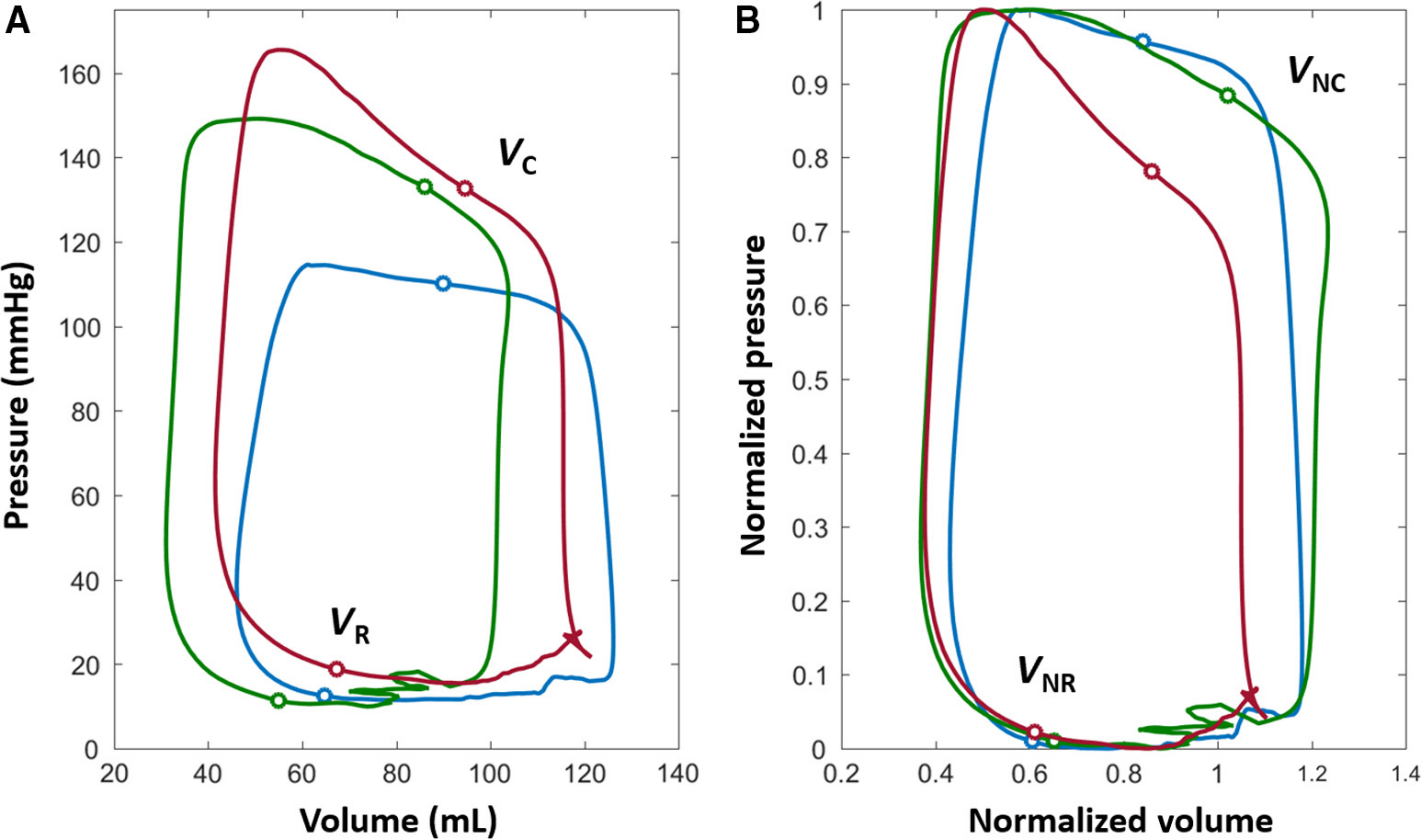
Comparison of conventional (A) and normalized (B) P‐V loops for three selected subjects. The inter‐subject variation in V_R_ is reduced to a localized region with normalization. The LV volume at the peak rate of chamber filling (V_NR_) has quantifiable load‐independent characteristics. See text for details.

Figure 4 depicts the conventional and normalized PVLs for multiple beats for a selected subject, illustrating the convergence of intra‐subject (beat‐to‐beat) variation in *V*_R_ with PVL normalization. Figure 5A shows individual, superimposed beats from three different subjects. Figure 5B shows the same inter‐subject beats in the nPVL, demonstrating the effect of normalization on reducing the inter‐subject variation in *V*_R_.

## Discussion

We introduce nPVL analysis to explore the existence of new load‐independent LV chamber properties. The key findings based on our preliminary data are: (1) the new method can reveal and quantify novel chamber properties at the organ system level; and (2) PVL normalization revealed that the volume at the peak rate of LV filling and, by analogy, the myocyte (sarcomere) length at the peak rate of myocyte (sarcomere) lengthening may possess load‐independent attributes. These preliminary findings support the conceptual framework provided by the nPVL loop method. This method has the potential to further characterize both cellular and macroscopic attributes.

### Normalization of LV pressure and volume

LV pressure normalization (0 ≤ *P*(t) ≤ 1 and −1 ≤ d*P*/d*t* ≤ 1) has been explored by Ghosh and Kovács (2013) in the quest for new load‐independent LV chamber properties. This led to the observation that peak −d*P*/dt, corresponding to the peak rate of cross‐bridge dissociation, is inscribed very close to 61% of the peak pressure of the previous cycle (*independent* of the peak pressure). In analogy to normalizing *P*(*t*) and d*P*/d*t*, it is natural to ask whether the normalization of LV volume can similarly reveal load‐independent LV chamber properties.

Accordingly, we introduce the nPVL. This entails the introduction of a *new method* for LV volume analysis by converting time‐varying volume into its dimensionless, normalized form. This is achieved by normalizing LV volume relative to the equilibrium (diastatic) volume (i.e., *V*_N_(*t*) = *V*(*t*)/*V*_diastasis_). This is the physiologically optimal method to normalize LV volume, since it normalizes all volume indices (e.g., stroke volume, end‐diastolic volume, and end‐systolic volume) with respect to the in‐vivo equilibrium LV volume without influencing LV ejection fraction. Therefore, all volume related features in systole and diastole can be quantified relative to the LV volume at diastasis.

Plotting normalized *P*(*t*) versus normalized *V*(*t*) generates the nPVL (Fig. 2), incorporating features found in both the normalized pressure phase‐plane and the normalized volume phase‐plane domains of physiologic hyperspace (Eucker et al. 2001, 2002).

### Physiological significance of LV equilibrium volume

Early rapid filling (Doppler E‐wave) is initiated by mechanical suction (d*P*/d*V* < 0), since the LV stores elastic strain energy when the end‐systolic volume is below the equilibrium (diastatic) volume (Shmuylovich et al. 2009). By focusing on kinematics (resultant motion), there is no requirement for intramyocardial stress–strain quantification to characterize LV mechanics, since the result of the complex, simultaneous intracellular and extracellular mechanisms boils down to the resultant kinematics—that is, the LV chamber (endocardial surface) expands at a faster rate than it can fill. Conceptually, the LV is an oscillatory ‘volume pump’ which oscillates relative to the equilibrium volume and is bounded by the end‐systolic and end‐diastolic volumes for each cardiac cycle. In sinus rhythm, this oscillation is both above and below the equilibrium volume (Fig. 1B). The magnitude of the oscillation above the equilibrium volume is determined by the Doppler A‐wave volume (Fig. 3). Naturally, during atrial fibrillation, the LV's volumetric oscillation is only below its equilibrium volume. The definition of the in‐vivo equilibrium volume has remained a topic of debate depending on whether a kinematic (Shmuylovich et al. 2009) or non‐kinematic (Remme et al. 2011) conceptual framework is employed.

### Load‐dependence of LV filling and ejection rates

The peak rate of myocyte shortening is known to be load‐dependent (McDonald et al. 1998); however, the load‐dependent attributes of the peak rate of myocyte lengthening relative to the unloaded (equilibrium) length have not been studied. On the cellular level, myocyte lengthening is a result of ATP‐dependent sarcoplasmic Ca^2+^ sequestration and cross‐bridge uncoupling (Bers 2000), allowing stored elastic strain from intracellular titin, extracellular connective tissue matrix, visceral pericardium, and load to lengthen the cell. Cross‐bridges start to uncouple after peak systolic pressure, and continue to uncouple during isovolumic relaxation and into early rapid filling. Cross‐bridge uncoupling is complete by the time diastasis is achieved. We found that the rate at which myocytes lengthen as a group can be further investigated by normalizing the PVL and studying the temporal derivatives of LV volume.

The peak rate of myocyte lengthening is analogous to the peak rate of early rapid filling (peak of Doppler E‐wave) and is inscribed at peak +d*V*/dt (Fig. 3). The LV volume at which peak +d*V*/d*t* occurs is denoted *V*_R_ and *V*_NR_ on the PVL and nPVL, respectively. By analogy, the peak rate of LV ejection during systole is defined as peak −dV/dt, and the LV volume at which peak −dV/dt occurs is denoted *V*_C_ and *V*_NC_ on the PVL and nPVL, respectively. When peak positive and peak negative d*V*/d*t* points are plotted on each PVL and nPVL, the normalized peak +d*V*/dt points converge to a localized intra‐subject (beat‐to‐beat) variation range of 9 ± 5%, revealing that normalization of *V*_R_ may exhibit quantifiable load‐independent properties (Fig. 4). In contrast, the normalized peak −d*V*/dt points do not tend to converge to a similar narrow range and exhibit an intra‐subject (beat‐to‐beat) variation of 17 ± 10%, suggesting that peak −d*V*/dt is unlikely to manifest load‐independent attributes. Furthermore, *V*_C_ is normalization insensitive, and is unlikely to be an important parameter for nPVL analysis.

### Intra‐subject (beat‐to‐beat) comparison of PVL and nPVL

Beat‐to‐beat variation in *V*_R_ in the conventional PVL was statistically reduced as compared to beat‐to‐beat variation in *V*_NR_ in the nPVL (13 ± 6% vs. 9 ± 5%, respectively; *P* = 0.0024). This reduction in *V*_R_ intra‐subject (beat‐to‐beat) variation with normalization suggests that *V*_NR_ may exhibit unique load‐independent properties (Fig. 4). Conversely, beat‐to‐beat variation in *V*_C_ in the conventional PVL showed no significant change as compared to beat‐to‐beat variation in *V*_NC_ in the nPVL (17 ± 9% vs. 17 ± 10%, respectively; *P* = 0.56). Thus, *V*_C_ did not demonstrate significant load‐independent attributes upon normalization and is likely of limited utility for characterizing load‐independent chamber properties in the normalized *P–V* plane.

### Inter‐subject comparison of PVL and nPVL

The inter‐subject variations in *V*_NR_ and *V*_NC_ were 8% and 11%, respectively (Table 3). The observed smaller inter‐subject variation in *V*_NR_ points to the existence of load‐independent properties related to myocyte lengthening, causing inter‐subject values of *V*_NR_ to be relatively constant (Fig. 5). In contrast, *V*_NC_ has a larger inter‐subject variation than *V*_NR_, indicating that the peak rate of myocyte shortening during ventricular ejection may be more load‐dependent than the peak rate of myocyte lengthening during early rapid filling (Doppler E‐wave).

Our results reveal that LV volume at the peak rate of chamber filling (peak of Doppler E‐wave) is generated at 64 ± 5% of the equilibrium (diastatic) LV volume. The small intra‐ and inter‐subject variation observed for V_NR_ in the 13 datasets and 161 PVLs studied provides compelling preliminary evidence that the dimensionless parameter defined by the LV volume at the Doppler E‐wave peak relative to *V*_diastasis_ has relatively load‐independent properties. At the cellular level, this observation suggests that myocyte (sarcomere) length at the peak rate of myocyte (sarcomere) lengthening relative to the resting myocyte (sarcomere) length may have load‐independent attributes.

### Ventriculo–arterial coupling

Because ventriculo–arterial (V–A) coupling is an effective index of the mechanical performance of the LV and of the dynamic modulation of the cardiovascular system (Chantler et al. 2008), we computed V–A coupling of single beats in conventional and normalized PVLs and found no difference between the conventional versus normalized data. This was predictable because of the algebraic relationship (shown below) that assures that V–A coupling is normalization independent:

$$\text{V‐A Coupling}=\frac{\text{ESV}-V_{0}}{\text{EDV‐ESV}}$$

$$\text{Normalized V‐A Coupling}=\frac{\frac{\text{ESV}}{V_{\mathrm{diastasis}}}-\frac{V_{0}}{V_{\mathrm{diastasis}}}}{\frac{\text{EDV}}{V_{\mathrm{diastasis}}}-\frac{\text{ESV}}{V_{\mathrm{diastasis}}}}=\text{V‐A Coupling}$$

### Translational potential

With the advent of new pharmacological and device based cardiovascular therapies such as biventricular synchronized pacing, transcatheter valve replacement and repair technologies, etc., there is a growing need to quantify the mechanistic consequences of beneficial or adverse LV remodeling at both the organ and cellular levels (Ten‐Brinke et al. 2010; Gaemperli et al. 2013). As such, a ‘new’ load‐independent parameter such as *V*_NR_ merits fuller assessment for its potential to quantitatively characterize LV remodeling consequences. Simultaneously recorded pressure–volume datasets are often collected as part of clinical study protocols, and can be utilized to generate nPVLs. Upon additional validation in other (human, animal) settings and pathologic datasets, the nPVL may aid in the investigation of new cardiovascular therapies and their physiologic impact on LV chamber properties in‐vivo.

### Study limitations

This preliminary analysis is limited by several factors. Most importantly, it is not intended to be a clinical study; hence, the reported analysis draws from a limited dataset of subjects with normal LV function with an unequal representation of age, gender, and race.

Although most (11 of 13) of the subjects were normotensive at the time of hemodynamic data acquisition (Table 4), the spectrum of antihypertensive medicines and other attributes (diabetes, ECG features, etc.) were not explicitly considered in this analysis. Additional work using a larger data set is justified to further characterize the reported findings of load‐independence of *V*_NR_ in human and animal datasets with and without pathophysiologic components. Some PVLs were excluded from this analysis due to excessive noise in the volume signal, thereby limiting the total number of subjects and cardiac cycles analyzed. In future investigations, correlation with the full spectrum of clinical variables can be assessed and may include PVL cycle efficiency as a measure to quantify the distortions in the shape of the PVLs and aid in appropriate loop selection. We also considered the end‐systolic and end‐diastolic *P–V* relationships and found that in light of the relatively narrow range of load variation observed among the beats analyzed—in comparison to, for example, inferior vena cava balloon inflation or other load variation maneuvers (i.e., volume loading)—rigorous comparison of these parameters in the normalized *P–V* plane could not be assured. Future projects where load variation is a primary goal to assess these types of relationships would provide the proper format for such a comparison.

## Conclusions

To advance the method for the elucidation and characterization of load‐independent LV chamber properties, we introduce the nPVL. Normalized, dimensionless pressure and volume axes are defined by 0 ≤ *P*(*t*) ≤ 1 and *V*_N_(*t*) = *V*(*t*)/*V*_diastasis_. We examined 13 pressure–volume datasets (normal physiology, 161 PVLs) and observed that the LV volume at the Doppler E‐wave peak (i.e., peak +d*V*/dt) was achieved at 64 ± 5% relative to the normalized equilibrium (diastatic) volume, and was essentially independent of end‐systolic and end‐diastolic volume. At the cellular level, these observations suggest that myocyte length at the peak rate of myocyte lengthening relative to the resting (diastatic) length may have load‐independent attributes. These preliminary results justify analysis in larger human and/or animal datasets to more fully assess the load‐dependence versus load‐independence of these and similar parameters of LV function.

## Conflict of Interest

The authors have no conflicts of interest.
